# Supplementary material for: Programmable RNA base editing with photoactivatable CRISPR-Cas13
Source: Nat Commun. 2024 Jan 22;15:673. doi: 10.1038/s41467-024-44867-2 (PMC10803366; doi:10.1038/s41467-024-44867-2)
Supplement: Supplementary file 1 — Supplementary Information [file 41467_2024_44867_MOESM1_ESM.pdf]

Supplementary Information

Programmable RNA base editing with photoactivatable CRISPR-Cas13

Jeonghye Yu[1], Jongpil Shin[1], Jihwan Yu[1], Jihye Kim[1], Daseuli Yu[2] & Won Do Heo[1,3]

[1]Department of Biological Sciences, Korea Advanced Institute of Science and Technology (KAIST), Daejeon, Republic of Korea

[2]Life Science Research Institute, KAIST, Daejeon, Republic of Korea

[3]KAIST Institute for the BioCentury (KIB), KAIST, Daejeon, Republic of Korea

Table of Contents

SUPPLEMENTARY TABLES

Supplementary Table 1
Supplementary Table 2
Supplementary Table 3
Supplementary Table 4
Supplementary Table 5
Supplementary Table 6

SUPPLEMENTARY FIGURES

Supplementary Figure 1
Supplementary Figure 2
Supplementary Figure 3
Supplementary Figure 4
Supplementary Figure 5
Supplementary Figure 6
Supplementary Figure 7
Supplementary Figure 8
Supplementary Figure 9
Supplementary Figure 10
Supplementary Figure 11
Supplementary Figure 12
Supplementary Figure 13
Supplementary Figure 14
Supplementary Figure 15

Supplementary Table 1: crRNA spacer sequences used for RNA knockdown.

Name	Spacer sequence
PspCas13b Non-Target crRNA	GTAATGCCTGGCTTGTCGACGCATAGTCTG
PspCas13b *λ2* crRNA	GTGATAAGTGGAATGCCATGACCGCTGTCA
PspCas13b *NRAS* crRNA	TCTTCAACACCCTGTCTGGTCTTGGCTGAG
PspCas13b *NFKB1* crRNA	CACCAGCTCTCTGACTGTACCCCCAGAGAC
PspCas13b *PPARG* crRNA	CGGCCTGTGGCATCCGCCCAAACCTGATGG
PspCas13b *KRAS* crRNA	GTATTATTTATGGCAAATACACAAAGAAAG
PspCas13b *PPIB* crRNA	gCAAAGATCACCCGGCCTACATCTTCATCTCCAATT CGTAGGTCAAAATAC
PspCas13b *STAT3* crRNA	gAGCATCACAATTGGCTCGGCCCCCATTCCCACA

Supplementary Table 2: Primers used for qPCR.

Target	Primer name	Sequence
Human *GAPDH*	hmGAPDH-For	CGCTCTCTGCTCCTCCTGTT
	hmGAPDH-Rev	CCATGGTGTCTGAGCGATGT
Human *NRAS*	hmNRAS-For	GTACCTATGGTGCTAGTGGG
	hmNRAS-Rev	CTGAGTCCCATCATCACTGC
Human *NFKB1*	hmNFKB1-For	AACTGGGCTACTCTGGCGCAG
	hmNFKB1-Rev	GGAGGCTGCCTGGATCACTTC
Human *PPARG*	hmPPARG-For	GTGATCTTAACTGTCGGATCC
	hmPPARG-Rev	ACAAATGTTTTGCCAGGGCC
Human *KRAS*	hmKRAS-For	TGCAATGAGGGACCAGTACA
	hmKRAS-Rev	TGAGCCTGTTTTGTGTCTACTG
Human *PPIB*	hmPPIB-For	AGATGAAGATGTAGGCCGGG
	hmPPIB-Rev	GATGCTCTTTCCTCCTGTGC
Human *STAT3*	hmSTAT3-For	GAATCCAACAACGGCAGCCT
	hmSTAT3-Rev	GGAGTGGGTCTCTAGGTCAA

Supplementary Table 3: Tiling mismatched crRNAs used to assess restoration of firefly luciferase activity by A-to-I RNA editing.

Name	Spacer sequence
PspCas13b Tiling 50nt 50 mismatch distance crRNA to target W417X	gCAGCCGTCCTTGTCGATGAGAGCGTTTGTAGCCTCGGGGTTGTTAACGTA
PspCas13b Tiling 50nt 48 mismatch distance crRNA to target W417X	GCCAGCCGTCCTTGTCGATGAGAGCGTTTGTAGCCTCGGGGTTGTTAACG
PspCas13b Tiling 50nt 46 mismatch distance crRNA to target W417X	gCAGCCAGCCGTCCTTGTCGATGAGAGCGTTTGTAGCCTCGGGGTTGTTAA
PspCas13b Tiling 50nt 44 mismatch distance crRNA to target W417X	gTGCAGCCAGCCGTCCTTGTCGATGAGAGCGTTTGTAGCCTCGGGGTTGTT
PspCas13b Tiling 50nt 42 mismatch distance crRNA to target W417X	gTGTGCAGCCAGCCGTCCTTGTCGATGAGAGCGTTTGTAGCCTCGGGGTTG
PspCas13b Tiling 50nt 40 mismatch distance crRNA to target W417X	GCTGTGCAGCCAGCCGTCCTTGTCGATGAGAGCGTTTGTAGCCTCGGGGT
PspCas13b Tiling 50nt 24 mismatch distance crRNA to target W417X	gAGTAGGCGATGTCGCCGCTGTGCAGCCAGCCGTCCTTGTCGATGAGAGCG
PspCas13b Tiling 50nt 22 mismatch distance crRNA to target W417X	gCCAGTAGGCGATGTCGCCGCTGTGCAGCCAGCCGTCCTTGTCGATGAGAG
PspCas13b Tiling 50nt 16 mismatch distance crRNA to target W417X	gCTCGTCCCAGTAGGCGATGTCGCCGCTGTGCAGCCAGCCGTCCTTGTCGA
PspCas13b Tiling 30nt 30 mismatch distance crRNA to target W417X	gCAGCCGTCCTTGTCGATGAGAGCGTTTGTA
PspCas13b Tiling 30nt 24 mismatch distance crRNA to target W417X	gTGCAGCCAGCCGTCCTTGTCGATGAGAGCG

PspCas13b Tiling 30nt 22 mismatch distance crRNA to target W417X	gTGTGCAGCCAGCCGTCCTTGTCGATGAGAG
PspCas13b Tiling 30nt 20 mismatch distance crRNA to target W417X	GCTGTGCAGCCAGCCGTCCTTGTCGATGAG
PspCas13b Tiling 30nt 16 mismatch distance crRNA to target W417X	gCGCCGCTGTGCAGCCAGCCGTCCTTGTCGA

Supplementary Table 4: Gene-specific reverse transcription primers.

**Gene**	**RT primer sequence**
Firefly luciferase W417X	GACCCCGGCGTCGAAGATGT

Supplementary Table 5: **crRNA spacer sequences used for RNA editing.**

Name	Spacer sequence
PspCas13b *Firefly* W417X crRNA	GATGTCGCCGCTGTGCAGCTAGCCGTCCTTGTCGAT
PspCas13b *KRAS* Y32C crRNA	GATCACATTCGTCCACAAAATGATTCTGAA
PspCas13b *PPIB* L162L crRNA	GCCAAACACCACATGCTTGCCATCcAGCCAGGCTGTCTTGACTGTCGTGA
PspCas13b *STAT3* Y705C crRNA	GGTCTTCAGGcATGGGGCAGCGCTACCTGG
PspCas13b *KRAS* D30D crRNA	GATCATATTCTTCCACAAAATGATTCTGAA
PspCas13b *CTNNB1* T41A crRNA	GAGGAGCUGUGUUAGUGGCACCAGAAUGGAU

Supplementary Table 6: Gene-specific PCR primers used for Sanger sequencing.

Target	Primer name	Sequence
Firefly luciferase W417X	Fluc_pcr-For	GGATGCTCTCCAGTTCGGCT
	Fluc_pcr-Rev	GGATGCTCTCCAGTTCGGCT
	Fluc-seq	GTCCGTGGCCCCATGATCA
KRAS Y32C and D30D	KRAS_pcr-For	AGAGAGGCCTGCTGAAAATGACTG
	KRAS_pcr-Rev	GTCCTCATGTACTGGTCCCTCA
	KRAS-seq	GTGGTAGTTGGAGCTGGTGG
PPIB L162L	PPIB_pcr-For	TCAAGGACTTCATGATCCAG
	PPIB_pcr-Rev	TACTCCTTGGCGATGGCAAA
	PPIB-seq	TCAAGGACTTCATGATCCAG
STAT3 Y705C	STAT3_pcr-For	CAGAGAGCCAGGAGCATCCTGA
	STAT3_pcr-Rev	TCTAAAGTGCGGGGGGACATCG
	STAT3-seq	TCTAAAGTGCGGGGGGACATCG

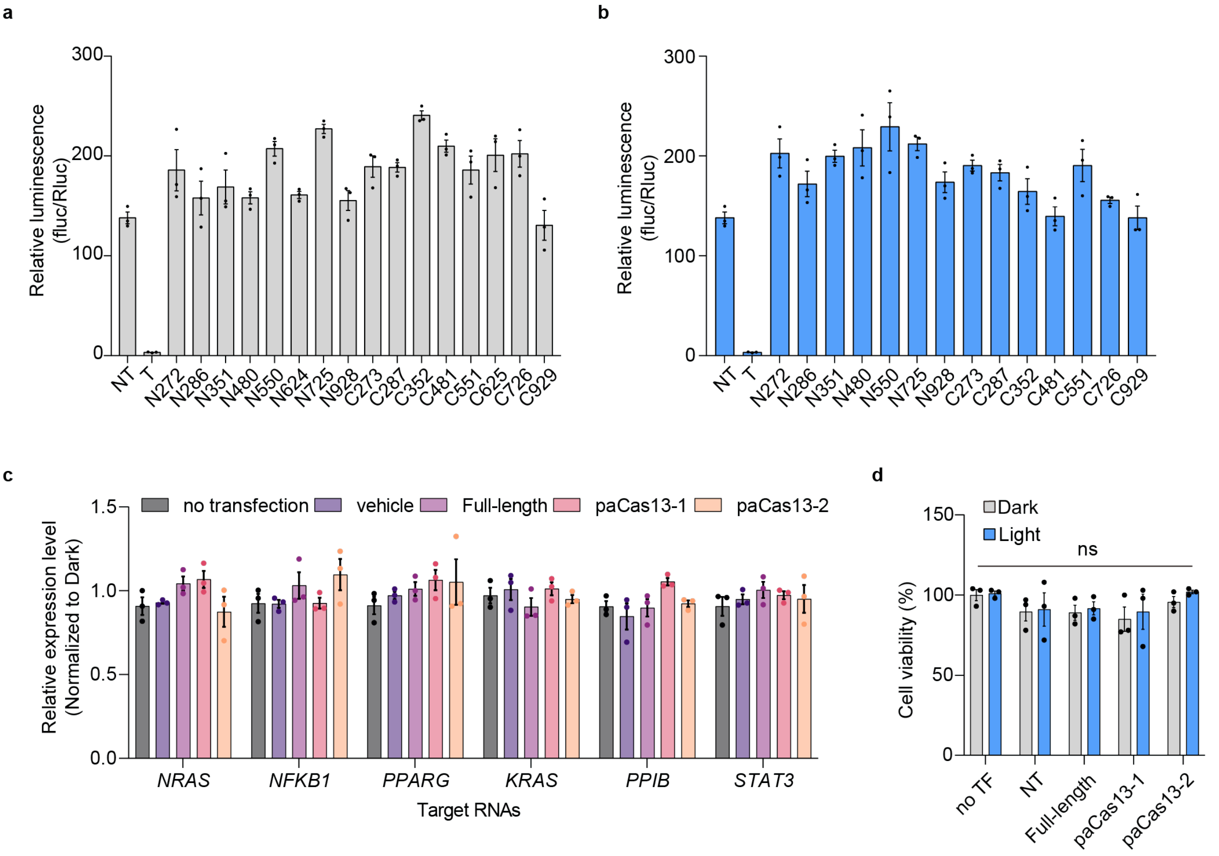

Supplementary Fig. 1: Screening background activity of N- and C-terminal fragments.

a, Background activity of the N- and C-fragment without an interaction partner. N-fragments fused with FRB and C-fragments fused with FKBP. **b,** Background activity of the N- and C-fragment without an interaction partner. N-fragments fused with pMag and C-fragments fused with nMagHigh1. **c,** Effect of light stimulation on RNA knockdown efficiency in HEK 293T cells transfected with full-length Cas13b or paCas13 candidates with targeted crRNAs. Data were normalized to corresponding groups in the dark condition. **d,** Cell viability effects of transfected constructs related to active Cas13 under dark and light conditions, as analyzed using two-way ANOVA. In all panels of this figure, n = 3 independent experiments. Error bars represent the mean ± s.e.m.

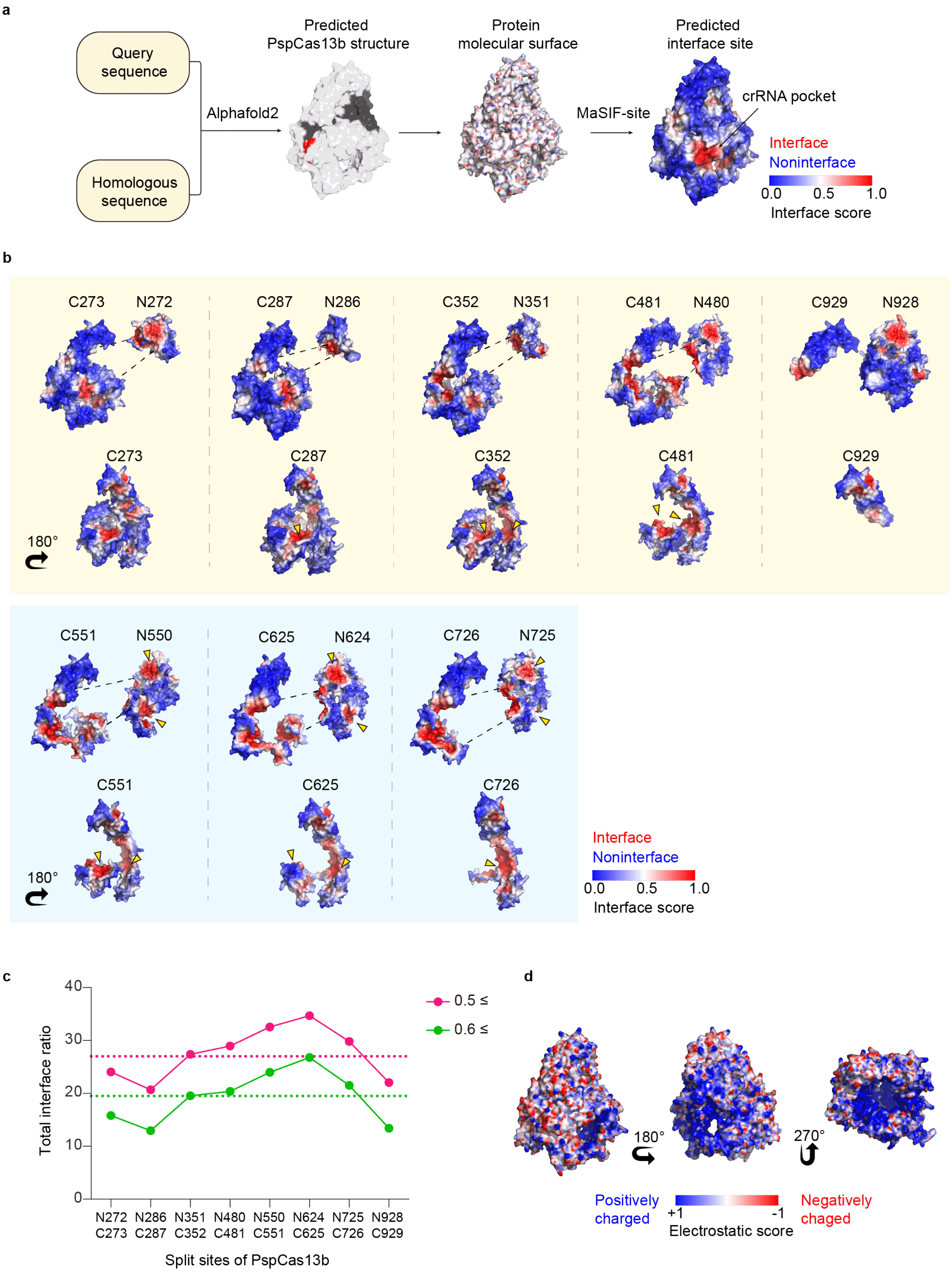

Supplementary Fig. 2: Computational prediction of characterization across the split Cas13 candidates.

a, Schematic representation of the prediction task workflow, from AlphaFold2 to MaSIF-site analysis. **b,** Visual comparison of split Cas13 candidates. Yellow triangles represent an interface; the yellow box represents split candidates without auto-assembly; and the blue box represents split candidates with auto-assembly. **c,** Quantification of interface properties across split sites. The utilized total interface ratio thresholds were 0.5 and above and 0.6 and above. The total interface ratio was calculated based on the number of atoms in each fragment exceeding the given score threshold, normalized by the total number of atoms in both. **d,** Electrostatic property of PspCas13b. Charge values above + 30 and below -30 were capped at those values and then normalized between -1 and 1.

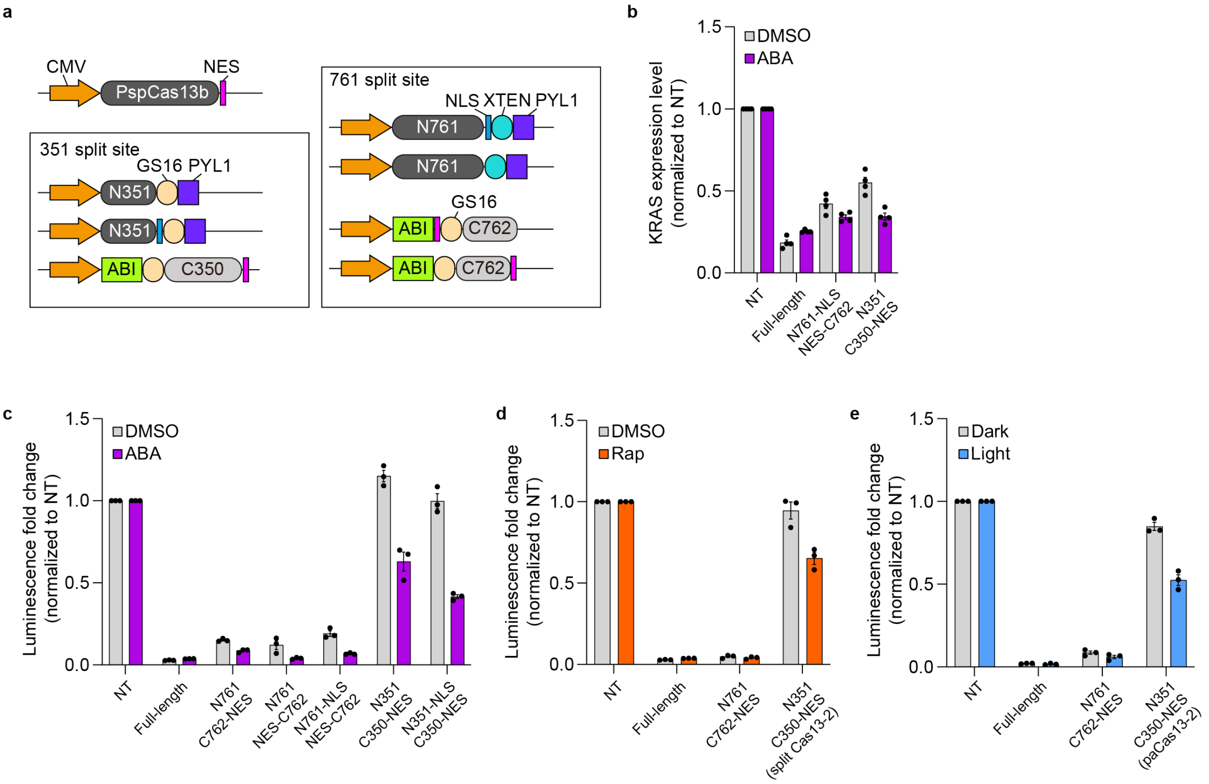

Supplementary Fig. 3: Comparative analysis of RNA degradation under various induction conditions.

a, Construction of full-length Cas13b and ABA-inducible system for comparative analysis. **b,** Endogenous KRAS expression level under 100 μM ABA induction. (n = 4 independent experiments) **c,** ABA-inducible RNA knockdown efficiency. **d,** Rapamycin-inducible RNA knockdown efficiency. **e,** Light-inducible RNA knockdown efficiency. For panels **c** and **d**, n = 3 independent experiments. Error bars represent the mean ± s.e.m.

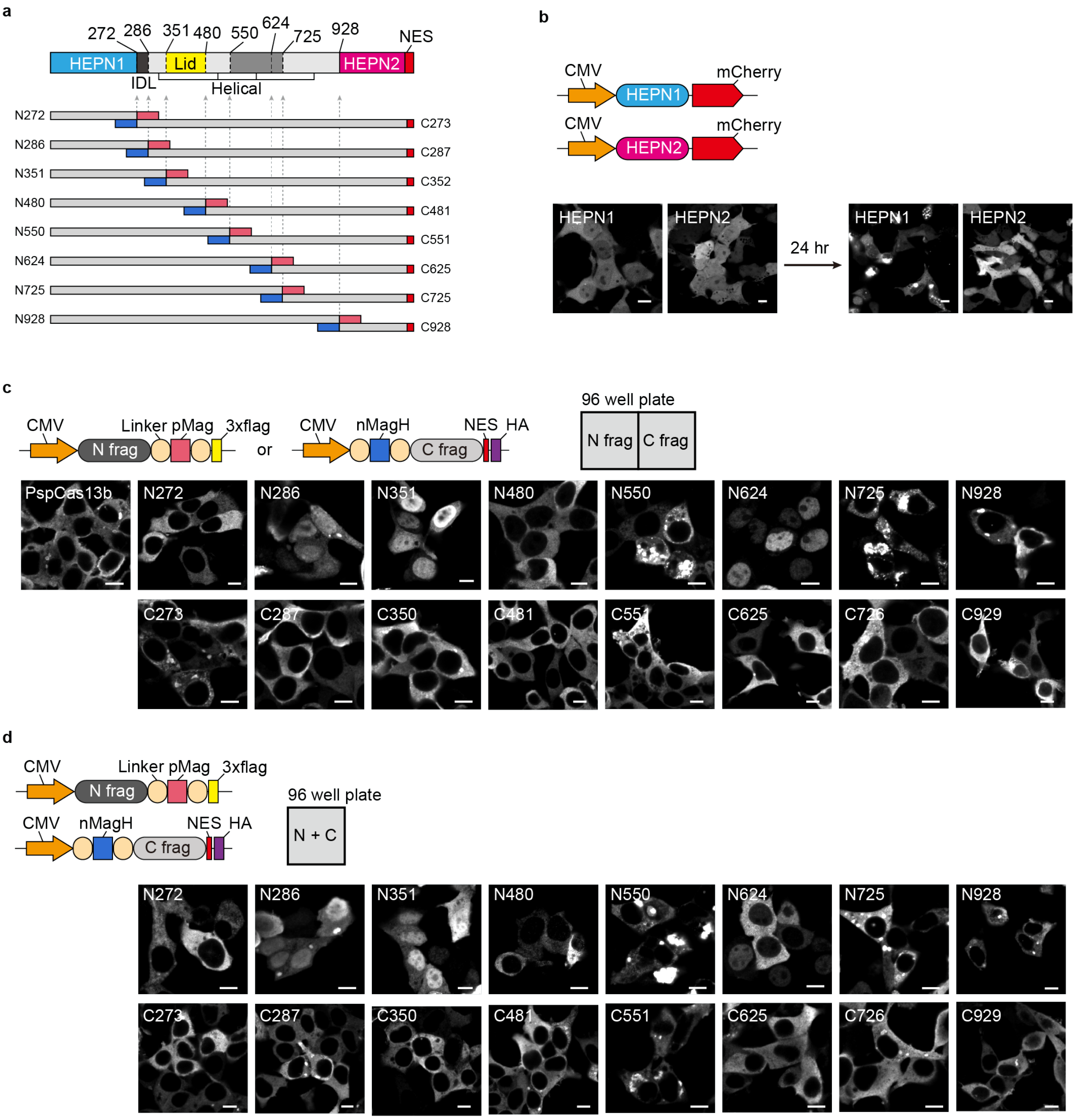

Supplementary Fig. 4: Subcellular localization of paCas13 fragments.

a, Schematic of the construct designs for the paCas13 fragments. **b,** Construction of HEPN domains with mCherry at the C-terminus. Representative 60X confocal microscopy images of HEK 293T cells are shown. Scale bar = 10 µm. **c,** Construction and expression of individual paCas13 fragments. Representative 60X confocal microscopy images of HEK 293T cells are shown. Scale bar = 10 µm. **d,** Co-expression of paCas13 fragments in the same well of a plate. Representative 60X confocal microscopy images of HEK 293T cells. Scale bar = 10 µm.

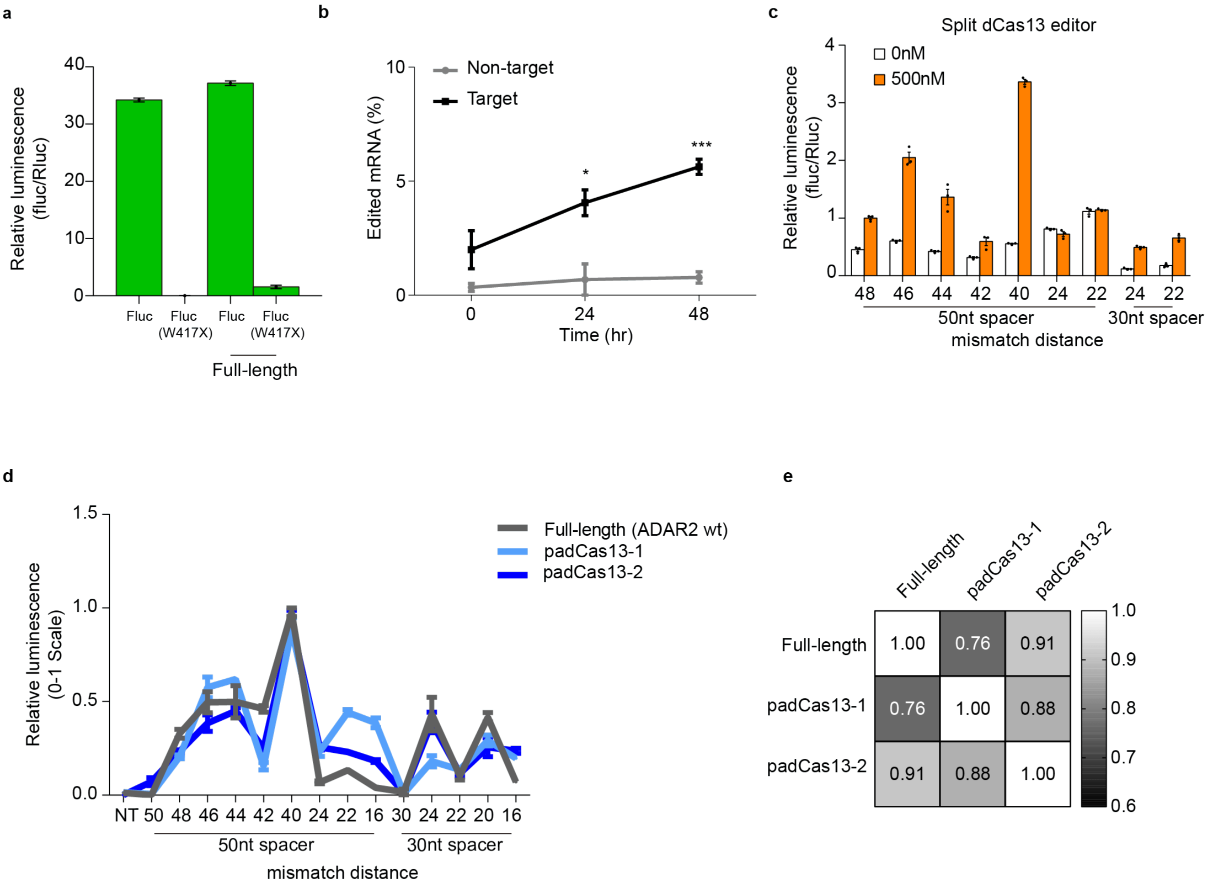

Supplementary Fig. 5: Characterization of the targeted luciferase reporter system.

a, Luciferase assay for firefly luciferase activity following transfection with W417X mutant of firefly luciferase (n = 3 independent experiments). **b,** Background editing effects under crRNA-only conditions (n = 3 independent experiments, *P = 0.0196 and ***P = 0.0003 by Student's two-tailed t-test). **c,** Restoration of chemical-induced luciferase activity via A-to-I RNA editing with tiled crRNAs in the presence of DMSO or rapamycin. Split dCas13 editor was coupled with $ADAR2_{DD}$ wild-type (n = 3 independent experiments). **d,** Normalized relative luminescence (scaled 0-1) representing RNA editing patterns across various tiling assays. **e,** Pearson r correlation analysis of RNA editing patterns derived from panel **d**. Error bars represent the mean ± s.e.m.

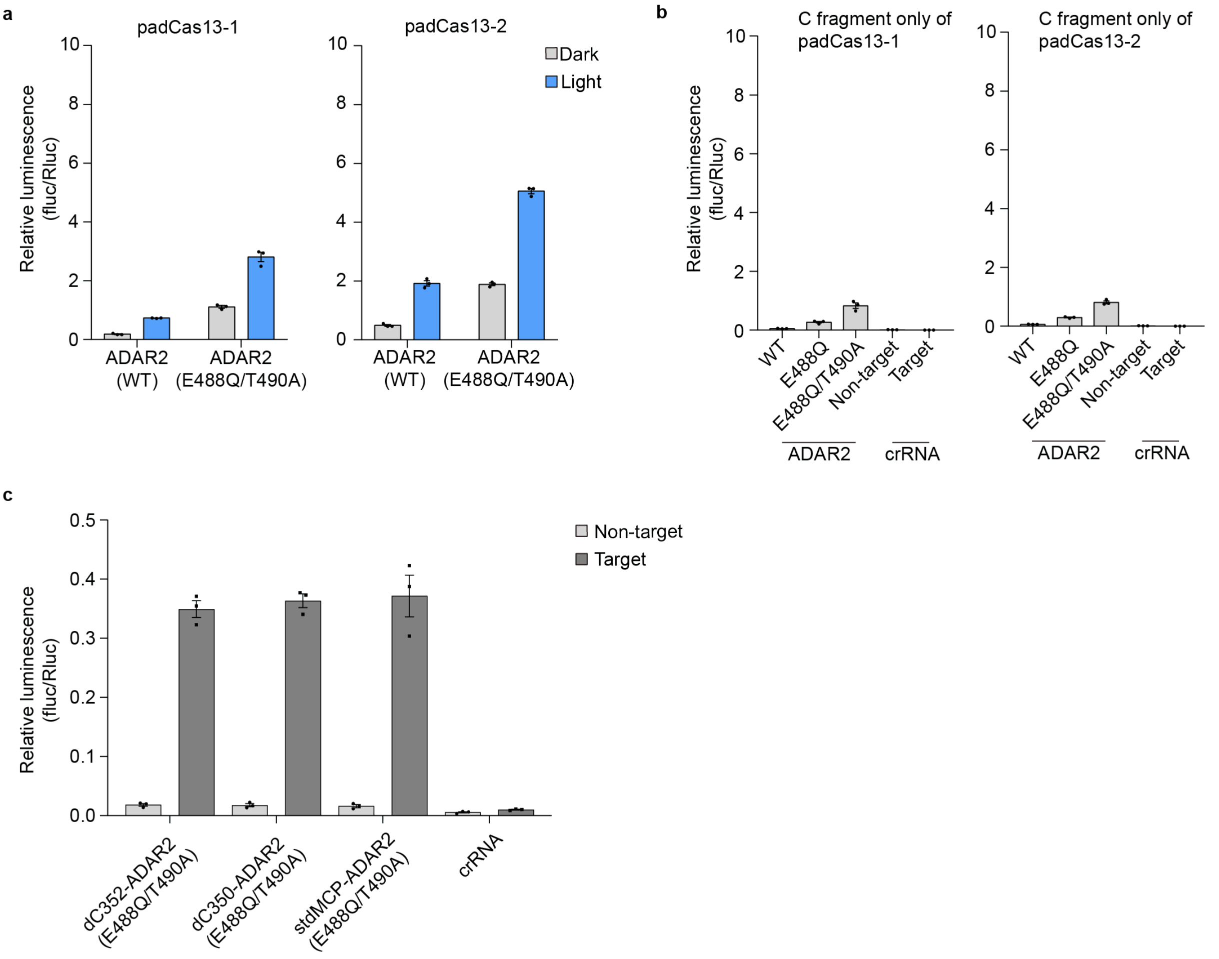

Supplementary Fig. 6: Characterization of a regulatory protein of the padCas13 system for RNA editing.

a, Restoration of light-induced luciferase activity via A-to-I RNA editing. Each padCas13 editor was coupled with wild-type or mutant ADAR2DD (E488Q/T490A). Blue light was delivered at 5-min intervals for 24 hours with an LED plate. **b,** Background effect of the padCas13 editor upon transfection of only the ADAR2DD-fused C-fragment or crRNA. **c,** Background effect of the ADAR2 fused proteins (C fragment of padCas13 editors and stdMCP) with crRNA. In all panels of this figure, n = 3 independent experiments. Error bars represent the mean ± s.e.m.

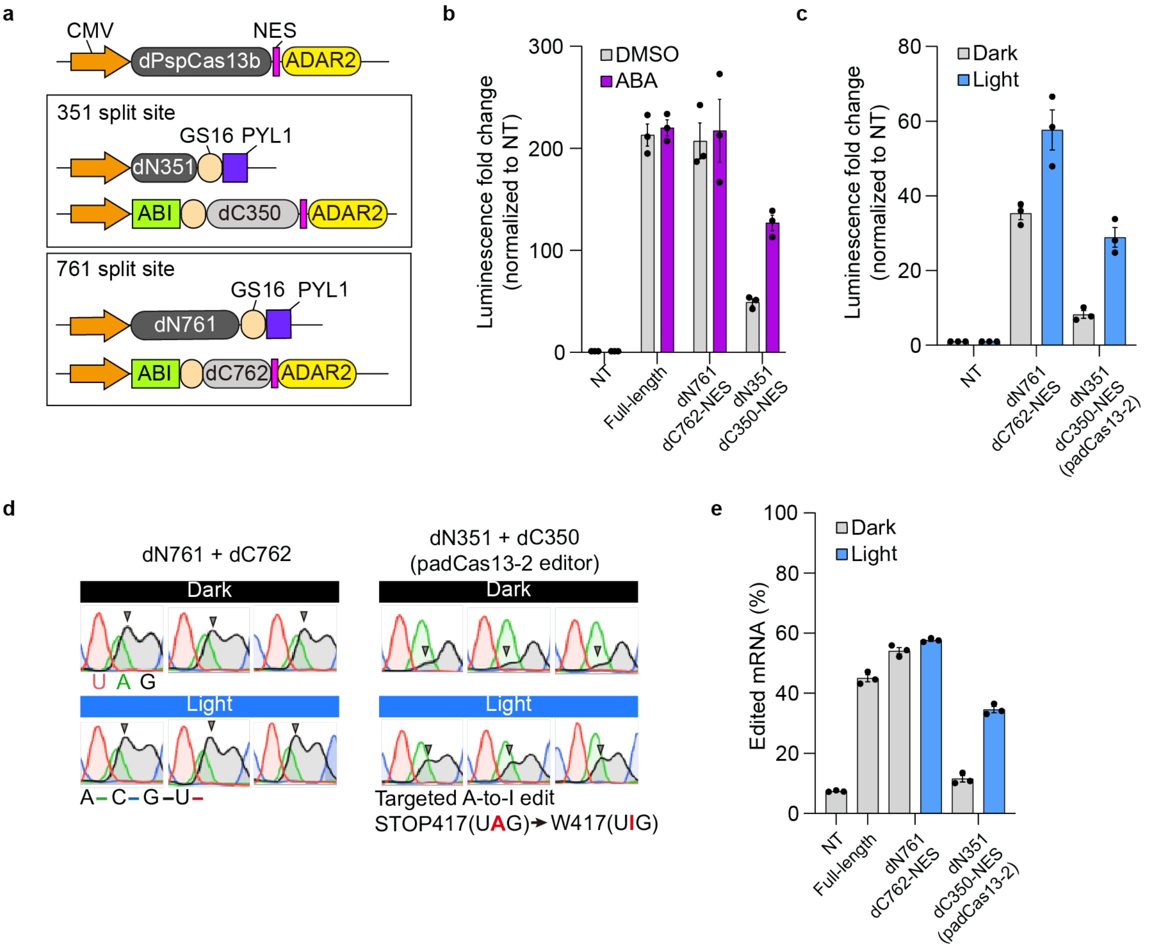

Supplementary Fig. 7: Comparative comparison of A-to-I RNA base editing under various induction conditions.

a, Construction of full-length Cas13b and ABA-inducible system for comparative RNA editing analysis. **b,** ABA-inducible luciferase restoration mediated by A-to-I RNA base editing under 100 μM ABA induction. **c,** Light-inducible luciferase restoration mediated by A-to-I RNA base editing. **d.** Chromatogram of RNA base editing at the targeted site. **e,** Quantitative assay of RNA editing levels shown in panel d. In all panels of this figure, n = 3 independent experiments. Error bars represent the mean ± s.e.m.

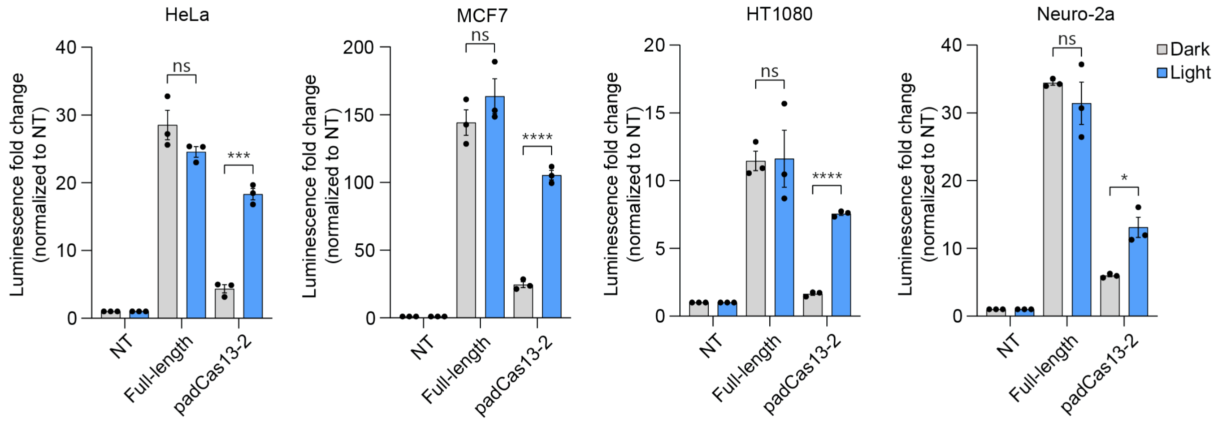

Supplementary Fig. 8 Optogenetic A-to-I RNA base editing of padCas13 editor in different cell lines.

HeLa, MCF7, HT1080, and Neuro-2a cells were transfected with a full-length Cas13b or padCas13-2 editor system with non-targeted (NT) or targeted crRNAs. All values were normalized to the corresponding NT crRNA group within each dark and light condition. The blue light was delivered at 5-min intervals for 24 hours with an LED plate (n = 3 independent experiments, *P = 0.0398, ***P = 0.0001, and ****P < 0.0001 by Student's two-tailed t-test). Error bars represent the mean ± s.e.m.

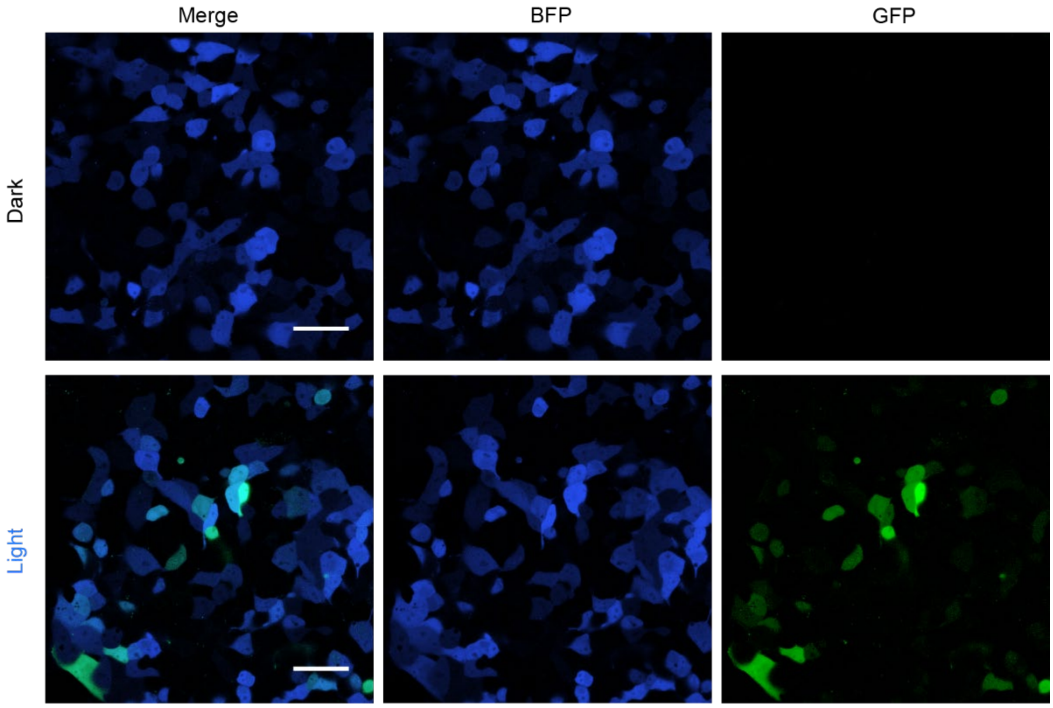

Supplementary Fig. 9: Representative images of GFP reporter by C-to-U RNA editing with the padCas13 editor.

Representative 60X confocal microscopy images of HEK 293T cells co-transfected with the padCas13-2 editor, the GFP reporter, and the target crRNA. Scale bar = 50 μm.

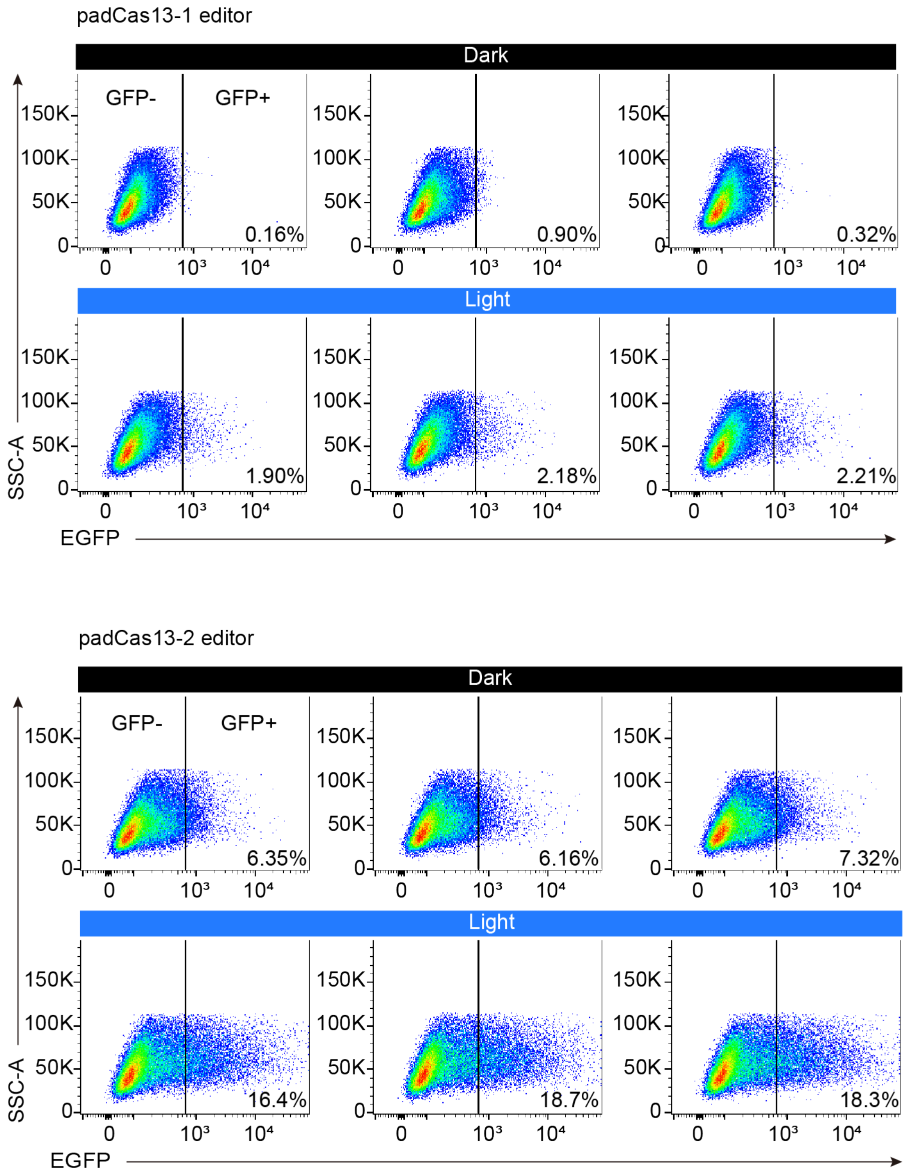

Supplementary Fig. 10: Representative images of GFP reporter by C-to-U RNA editing with padCas13 editor.

Flow cytometry plots of cells transfected with the padCas13 editors with cytidine deaminase. FITC-A (log scale) is shown on the X-axis; SSC-A (linear scale) is shown on the Y-axis.

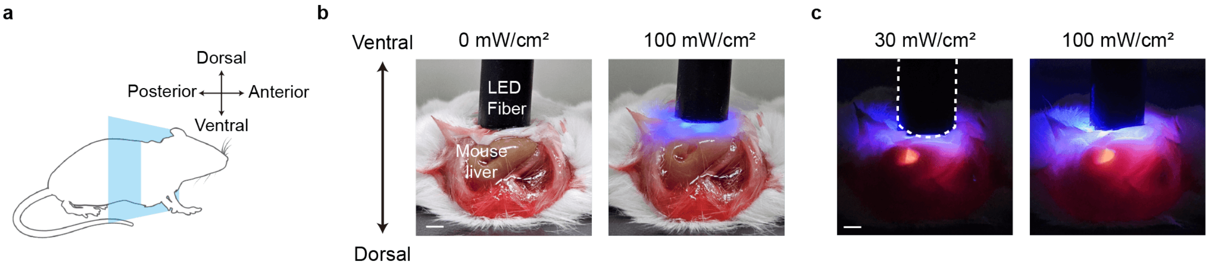

Supplementary Fig. 11: Blue light penetration in a transverse mouse section.

a, Schematic illustration detailing the position from which we obtained the transverse section of the mouse (6-week-old Balb/c). **b**, Anterior view of the dissected abdomen positioned under the fiber-type blue LED core (Ø 6 mm, 470nm). Blue LED was delivered to the abdomen skin of the mouse. **c**, Comparison of blue light penetration to the liver under varying light conditions. Scale bar = 2 mm.

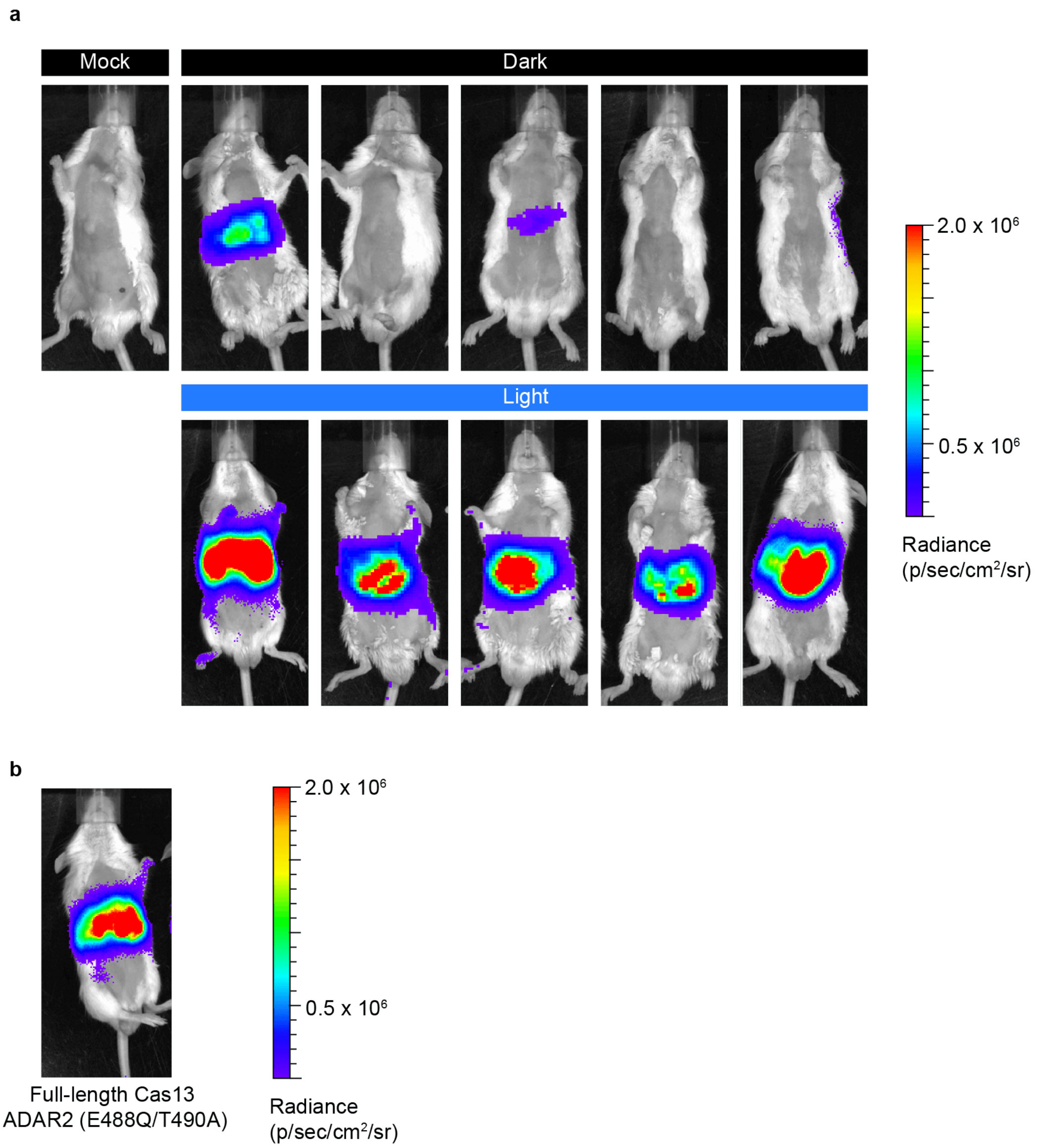

Supplementary Fig. 12: Representative images of in vivo RNA editing by the padCas13 editor.

a, Luminescence images of mice carrying a luciferase reporter (W417X) and the padCas13-2 editor. Only the reagent is delivered under the mock condition. **b,** Luminescence images of mouse carrying a full-length Cas13b fused with ADAR2$_{DD}$ (E488Q/T490A).

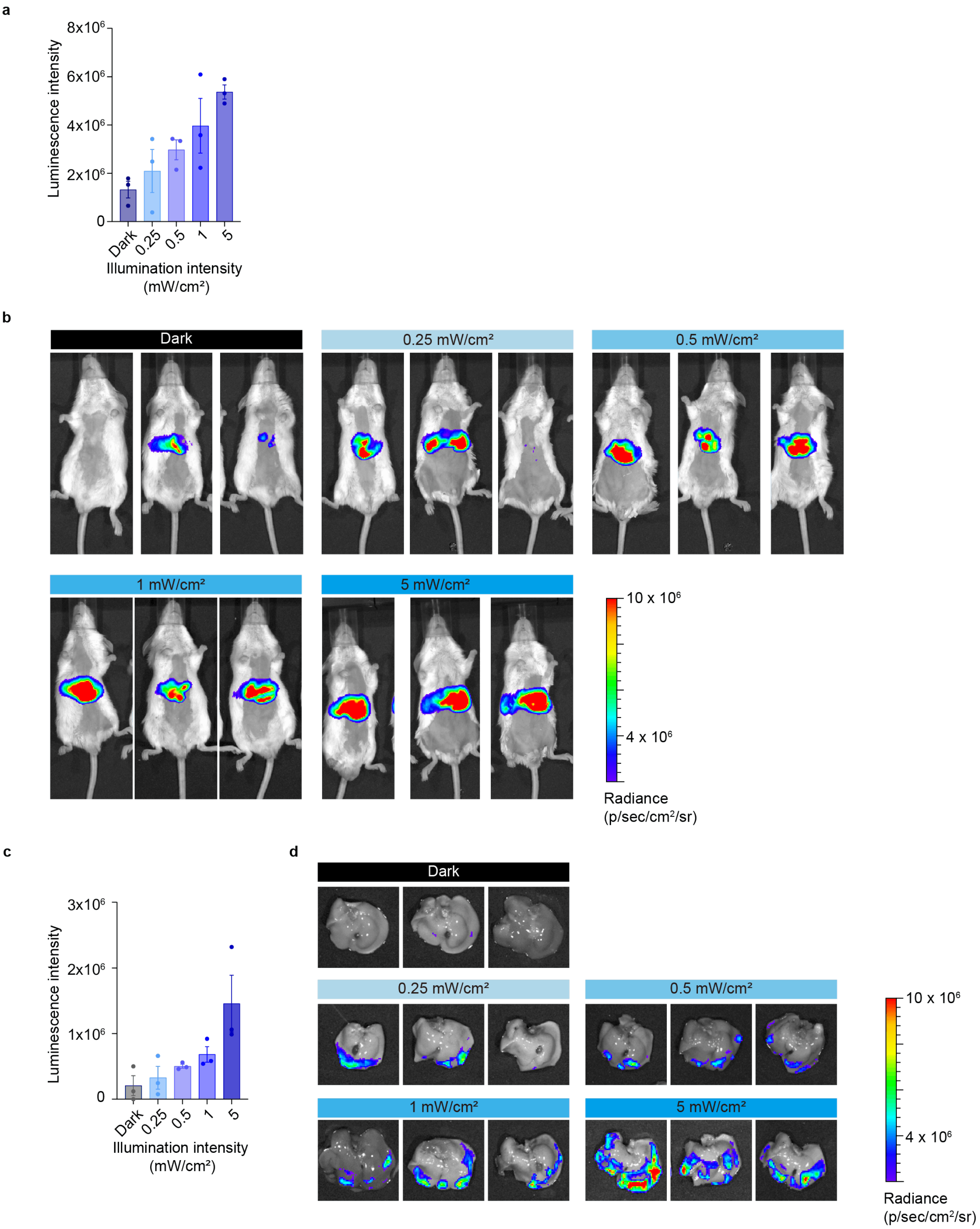

Supplementary Fig. 13: Light-dependent padCas13 editor activity in vivo and isolated mouse livers.

a, Intensity-dependent padCas13 editor activity measured from mice in vivo. **b**, Luminescence images of the padCas13 editor system-carrying mice used to generate the data presented in panel (**a**). **c**, Intensity-dependent padCas13 editor activity of liver tissues isolated from the transfected mice shown in panel (**b**). **d**, Luminescence images of the isolated liver tissues used to generate the data presented in panel (**c**). Error bars represent the mean ± s.e.m.

a

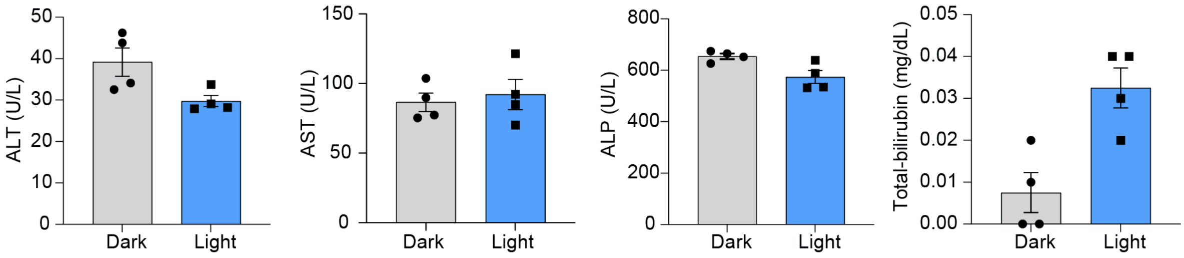

b

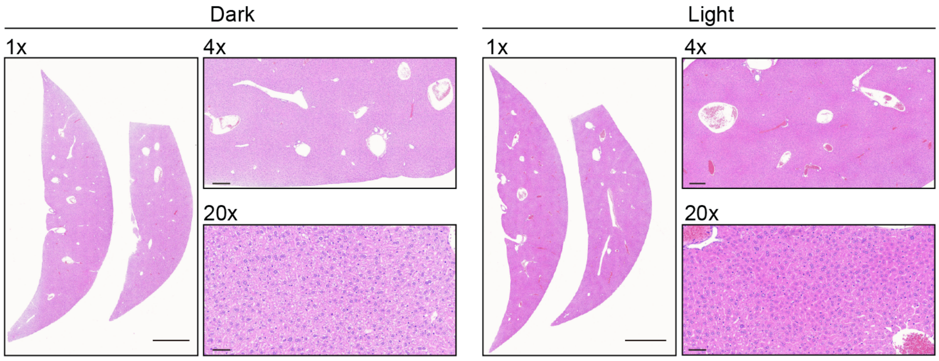

Supplementary Fig. 14: Comparative analysis of liver damage under dark and light exposure in mice.

a, Biochemical analysis of blood samples from livers of mice held under dark and light conditions, assessing levels of alanine aminotransferase (ALT), aspartate aminotransferase (AST), alkaline phosphatase (ALP), and total bilirubin (n = 4 mice per group). Error bars represent the mean ± s.e.m. **b,** Histological analysis of hematoxylin and eosin (H&E) stained livers. Images are presented at magnifications of 1x (scale bar = 2 mm), 4x (scale bar = 300 μm), and 20x (scale bar = 60 μm).

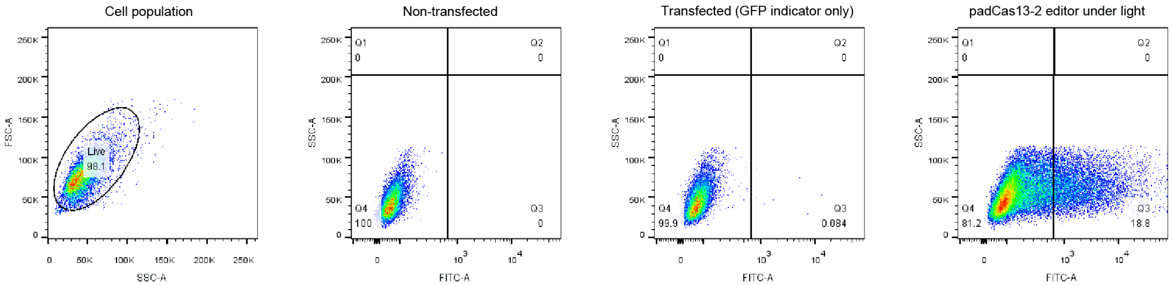

Supplementary Fig. 15: Gating strategy for flow cytometry experiments.

Gating strategy for Fig. 4e. SSC-A/FSC-A to gate living cells. A population of living cells was analyzed for GFP (FITC-A). The percentage of GFP+ fluorescence cells is Q3.
