# Supplementary material for: Programmable RNA base editing with photoactivatable CRISPR-Cas13
Source: Nat Commun. 2024 Jan 22;15:673. doi: 10.1038/s41467-024-44867-2 (PMC10803366; doi:10.1038/s41467-024-44867-2)
Supplement: Supplementary file 2 — Reporting Summary [file 41467_2024_44867_MOESM2_ESM.pdf]

nature portfolio

Corresponding author(s): Won Do Heo

Last updated by author(s): Dec 23, 2023

Reporting Summary

Nature Portfolio wishes to improve the reproducibility of the work that we publish. This form provides structure for consistency and transparency in reporting. For further information on Nature Portfolio policies, see our Editorial Policies and the Editorial Policy Checklist.

Statistics

For all statistical analyses, confirm that the following items are present in the figure legend, table legend, main text, or Methods section.

n/a	Confirmed	
☐	☒	The exact sample size (n) for each experimental group/condition, given as a discrete number and unit of measurement
☐	☒	A statement on whether measurements were taken from distinct samples or whether the same sample was measured repeatedly
☐	☒	The statistical test(s) used AND whether they are one- or two-sided<br>*Only common tests should be described solely by name; describe more complex techniques in the Methods section.*
☒	☐	A description of all covariates tested
☒	☐	A description of any assumptions or corrections, such as tests of normality and adjustment for multiple comparisons
☐	☒	A full description of the statistical parameters including central tendency (e.g. means) or other basic estimates (e.g. regression coefficient) AND variation (e.g. standard deviation) or associated estimates of uncertainty (e.g. confidence intervals)
☐	☒	For null hypothesis testing, the test statistic (e.g. F, t, r) with confidence intervals, effect sizes, degrees of freedom and P value noted<br>*Give P values as exact values whenever suitable.*
☒	☐	For Bayesian analysis, information on the choice of priors and Markov chain Monte Carlo settings
☒	☐	For hierarchical and complex designs, identification of the appropriate level for tests and full reporting of outcomes
☐	☒	Estimates of effect sizes (e.g. Cohen's d, Pearson's r), indicating how they were calculated

Our web collection on statistics for biologists contains articles on many of the points above.

Software and code

Policy information about availability of computer code

Data collection	Tecan spark 10M microplate reader is used for dual-luciferase reporter assay. Bio-rad CFXMaestro 96 instrument is used for real-time PCR. BD LSRFortessa is used for flow cytometry. Nikon A1R confocal microscope mounted on a Nikon Eclipse Ti body with a Nikon CFI Plan Apochromat VC 60X/1.4 numerical aperture (NA) and NIS-elements AR 64-bit version 3.21 are used for fluorescence image. IVIS Lumina xenogen is used for in vivo luciferase image.
Data analysis	Protein structure was predicted using Alphafold2 and analyzed in PyMOL 2.5.2.<br>Data was analyzed in Graphpad Prism 7 and Microsoft Excel. Imaging data was analyzed in Nikon imaging software 5.21. in vivo date was analyzed in PerkinElmer Living Image Software 2.6. and Image Studio software 5.2.

For manuscripts utilizing custom algorithms or software that are central to the research but not yet described in published literature, software must be made available to editors and reviewers. We strongly encourage code deposition in a community repository (e.g. GitHub). See the Nature Portfolio guidelines for submitting code & software for further information.

Data

Policy information about availability of data

All manuscripts must include a data availability statement. This statement should provide the following information, where applicable:

- Accession codes, unique identifiers, or web links for publicly available datasets
- A description of any restrictions on data availability
- For clinical datasets or third party data, please ensure that the statement adheres to our policy

All data supporting the findings of this study are available in the paper.

Human research participants

Policy information about studies involving human research participants and Sex and Gender in Research.

Reporting on sex and gender	N/A
Population characteristics	N/A
Recruitment	N/A
Ethics oversight	N/A

Note that full information on the approval of the study protocol must also be provided in the manuscript.

Field-specific reporting

Please select the one below that is the best fit for your research. If you are not sure, read the appropriate sections before making your selection.

[x] Life sciences [ ] Behavioural & social sciences [ ] Ecological, evolutionary & environmental sciences

For a reference copy of the document with all sections, see nature.com/documents/nr-reporting-summary-flat.pdf

Life sciences study design

All studies must disclose on these points even when the disclosure is negative.

Sample size	All experiments shown were conducted with at least 3 independent experiments. Regarding image data, at least 86 cells were used for imaging analysis.
Data exclusions	No data was excluded.
Replication	For each experiment, all data is representative of duplicates or triplicates.
Randomization	Cells and mice were randomized to experimental groups.
Blinding	All groups were not blinded as sample preparation followed uniform protocols. This is not relevant to biological experiments and samples were not blinded

Reporting for specific materials, systems and methods

We require information from authors about some types of materials, experimental systems and methods used in many studies. Here, indicate whether each material, system or method listed is relevant to your study. If you are not sure if a list item applies to your research, read the appropriate section before selecting a response.

Materials & experimental systems

n/a	Involved in the study	
☐	☒	Antibodies
☐	☒	Eukaryotic cell lines
☒	☐	Palaeontology and archaeology
☐	☒	Animals and other organisms
☒	☐	Clinical data
☒	☐	Dual use research of concern

Methods

n/a	Involved in the study	
☒	☐	ChIP-seq
☐	☒	Flow cytometry
☒	☐	MRI-based neuroimaging

Antibodies

Antibodies used	anti-HA (C29F4) rabbit antibody: Cell Signaling Technology (#3724)<br>anti- DYKDDDDK Tag (D6W5B) rabbit antibody: Cell Signaling Technology (#14793)<br>anti-rabbit IgG (H+L) Highly cross-adsorbed goat Secondary Antibody, Alexa Fluor 488: Invitrogen (A-11034)
Validation	Antibodies were only chosen if there were validated references available.

Eukaryotic cell lines

Policy information about cell lines and Sex and Gender in Research

Cell line source(s)	HEK 293T (CRL-11268), HeLa (CCL-2), MCF7 (HTB-22), HT1080 (CCL-121), and Neuro-2a (CCL-131) cell lines were acquired from ATCC.
Authentication	None of the cell lines used were authenticated.
Mycoplasma contamination	The cell line tested negative for mycoplasma contamination.
Commonly misidentified lines (See ICLAC register)	No commonly misidentified cell lines were used.

Animals and other research organisms

Policy information about studies involving animals; ARRIVE guidelines recommended for reporting animal research, and Sex and Gender in Research

Laboratory animals	Six-week-old male BALB/cAnNTac mice were purchased from Raonbio in South Korea.
Wild animals	There were no wild animals used in this study.
Reporting on sex	male mice
Field-collected samples	No field-collected samples were used in this
Ethics oversight	Animal experiments and treatments followed the guidelines of the Institutional Animal Care and Use Committees (IACUC) at Korea Advanced Institute of Science and Technology (KAIST). The approval number for this study is KA2023-089-v1.

Note that full information on the approval of the study protocol must also be provided in the manuscript.

Flow Cytometry

Plots

Confirm that:

☒ The axis labels state the marker and fluorochrome used (e.g. CD4-FITC).

☒ The axis scales are clearly visible. Include numbers along axes only for bottom left plot of group (a 'group' is an analysis of identical markers).

☒ All plots are contour plots with outliers or pseudocolor plots.

☒ A numerical value for number of cells or percentage (with statistics) is provided.

Methodology

Sample preparation	HEK293T cells in a 6-well plate were detached with trypsin and resuspended in an ice-cold FACS buffer.
Instrument	BD LSRFortessa flow cytometer

Software	BD FACSDiva software
Cell population abundance	Approximately 90% of cells are sorted from the living.
Gating strategy	Cells are gated by SSC-A/FSC-A in order to gate living cells. A population of living cells was analyzed for GFP (FITC-A).

☒ Tick this box to confirm that a figure exemplifying the gating strategy is provided in the Supplementary Information.
